# Small bites for big problems: stepwise aggregate degradation by autophagy

**DOI:** 10.1042/BST20250460

**Published:** 2026-06-22

**Authors:** Mark S. Hipp, Mario Mauthe

**Affiliations:** 1 Department of Biomedical Sciences, University of Groningen, University Medical Center Groningen, Groningen, The Netherlands; 2 School of Medicine and Health Sciences, Carl von Ossietzky University Oldenburg, Oldenburg, Germany

**Keywords:** autophagy, cellular protein quality control, molecular chaperones, piecemeal, selective autophagy receptors

## Abstract

Protein aggregates are a pathological hallmark of diverse disorders, including many neurodegenerative diseases, but also cardiometabolic disease and cancer. While the ubiquitin–proteasome system efficiently removes many soluble misfolded proteins, large or persistent assemblies often require the autophagy–lysosome pathway for their degradation. In the present mini-review, we summarize our knowledge of aggrephagy, the selective clearance of protein aggregates by autophagy, and discuss two recent manuscripts that argue that some aggregates must be primed for autophagosomal degradation, through chaperone-mediated remodeling. Aggrephagy substrates are defined by aggregate architecture, biophysical state, surface accessibility, and the physical constraints of membrane capture. These features help to explain why recruitment of selective autophagy receptors is necessary yet insufficient for clearance. Receptor clustering is required to concentrate early autophagy factors to establish initiation hubs, but successful degradation often requires upstream generation of smaller ‘aggrephagy-competent’ cargo units, which contain autophagy receptor clusters that successfully initiate autophagosome formation. Recent work supports a model in which larger aggregates are cleared through stepwise degradation enabled by prior remodeling steps that involve p97/VCP-driven disintegration or a chaperone module (DNAJB6–HSP70–HSP110) cooperating with the proteasomal 19S regulatory particle.

## Introduction

Protein aggregates are a hallmark of many degenerative diseases and are considered to be major drivers or contributors to cellular toxicity [1]. Most prominently, in neurodegenerative diseases the process of aggregate formation is considered to play a large role in the pathology of Huntington’s, Alzheimer’s, and Parkinson’s disease as well as other forms of dementia [2,3]. Although the precise role that aggregates play in each type of neurodegenerative disease is not fully understood, generally they are considered to damage lipid membranes, sequester proteins and render them non-functional, overload the protein quality control (PQC) system (e.g. chaperones, ubiquitin–proteasome system (UPS), and autophagy) [4–6], or to cause organelle stress, all of which can lead to cellular toxicity [5,7]. Besides their prominent role in neurodegenerative diseases, protein aggregates are also observed in other disease types. In cardiovascular diseases, aggregates have been associated with cardiac failure [8,9], in diabetes with ineffective insulin secretion [10,11], and in kidney disease models an increased number of aggregates has been detected, which may contribute to renal fibrosis [12–14]. In cancer, protein aggregation, particularly that of tumor suppressor proteins such as p53 and axin, plays a significant role in oncogenesis [15–17].

Because of the detrimental effects of the intracellular protein aggregates, it is crucial for cells to prevent their accumulation. Central to this process are the molecular chaperones, which facilitate initial protein folding after translation or re-folding after cellular stresses, such as heat stress [18]. If folding is unsuccessful, cells utilize two principal degradation routes to clear misfolded or unwanted proteins before they can aggregate: the UPS and autophagy [19]. How the PQC system handles aggregates after they have formed is much less understood and is the main subject of this review.

The UPS is the primary pathway for the regulated turnover of short-lived proteins but is also involved in the degradation of misfolded or otherwise damaged proteins [20]. Its role in aggregate reduction is therefore mainly to degrade misfolded proteins before they aggregate. Proteins are targeted for proteasomal degradation through the ATP-dependent attachment of poly-ubiquitin chains through E3-ligases [21]. Degradation of proteins by the proteasome requires that proteins pass a narrow opening that controls the access to the central cavity, which allows entry only to unfolded substrates [22]. This process is enabled by a ring of six ATPases as part of the 19S regulatory particle (19S-RP), which is able to unfold most proteins [23].

In contrast, autophagy delivers its cargo to the lysosomes without the need to first unfold it completely. Once cargo (including aggregates) is successfully delivered to the interior of a lysosome, catabolic enzymes such as proteases, lipases, and acidic hydrolases are able to degrade it [24,25]. In mammalian cells, there are three distinct autophagy pathways: chaperone-mediated autophagy (CMA), microautophagy, and macroautophagy. In CMA, single proteins that have a specific HSC70 binding motif are transported to the lysosomes by the chaperone HSC70 and are funneled into the lysosomes via the specific lysosomal transporter LAMP2A [26]. Although CMA cannot directly degrade protein aggregates, inhibition of CMA can lead to increased protein aggregates, because of its involvement in the removal of soluble proteins that would result in aggregates [27,28]. In microautophagy, cargo is directly sequestered by either late endosomes or lysosomes [29]. During macroautophagy (hereafter called autophagy), cargo is sequestered into a double-membrane vesicle, called an autophagosome, that subsequently fuses with lysosomes for cargo breakdown. There are in principle two ways of cargo collection by autophagy; non-selective bulk autophagy and a selective form of autophagy (see the ‘Aggrephagy: selective autophagic degradation of protein aggregates’ section) [30,31]. Non-selective autophagy can be triggered by nutrient starvation or a variety of cellular forms of stress (e.g. hypoxia, glucose/energy starvation, metabolic changes) to maintain cellular homeostasis [32]. Autophagy initiation and autophagosome biogenesis are typically triggered by nutrient or energy stress that inhibits mTORC1 and activates AMPK [33]. The *de novo* generation of autophagosomes relies on the action of the autophagy-related (ATG) proteins, which work in functional clusters. The ULK1 kinase complex initiates phagophore formation by phosphorylating downstream factors and by functioning as a scaffold [34]. The class III phosphatidylinositol 3-kinase complex I generates PI3P and recruits PI3P-binding effectors such as WIPI proteins and DFCP1, helping to organize the growing phagophore [34]. In parallel, ATG9-positive vesicles along with other vesicles that originate from multiple membrane sources fuse to successfully initiate phagophore nucleation [34,35]. Phagophore expansion and eventual closure depend on coordinated action of the ATG2–WIPI module and two ubiquitin-like conjugation systems [34,36]: the ATG12 system and the LC3/ATG8 system. These pathways mediate the conjugation of ATG8 family members to phosphatidylethanolamine on the growing phagophore membrane [34,36,37]. Lipid supply for expansion of the phagophore is driven by ATG2 proteins, aided by WIPI4 and ATG9A, enabling direct lipid transfer from the ER [38]. Autophagosome tethering and fusion with lysosomes is coordinated by ATG14, different Rab proteins and specific SNARE proteins [39,40].

The large size of the autophagosome [41] enables the degradation of larger intracellular material, including damaged organelles and aberrant protein assemblies [21,24]. Consequently, autophagy, and to a lesser extent microautophagy [29,42,43], has been attributed to degradation of protein aggregates, which is why stimulation of autophagy is considered a potential drug target to battle aggregate-associated neurodegeneration [44–46]. The vital physiological function of protein aggregate degradation by autophagy is best illustrated in animal models and patients, in which the loss of *ATG* genes results in increased accumulation of protein aggregates in various organs [47–51]. Moreover, neuronal *ATG* gene depletion (e.g. ATG5, ATG7, FIP200, WDR45/WDR45B) is sufficient to cause neurodegeneration-associated behavior in animal models [47–51].

## Main

### Protein aggregates and disaggregation machineries

**Figure 1:**
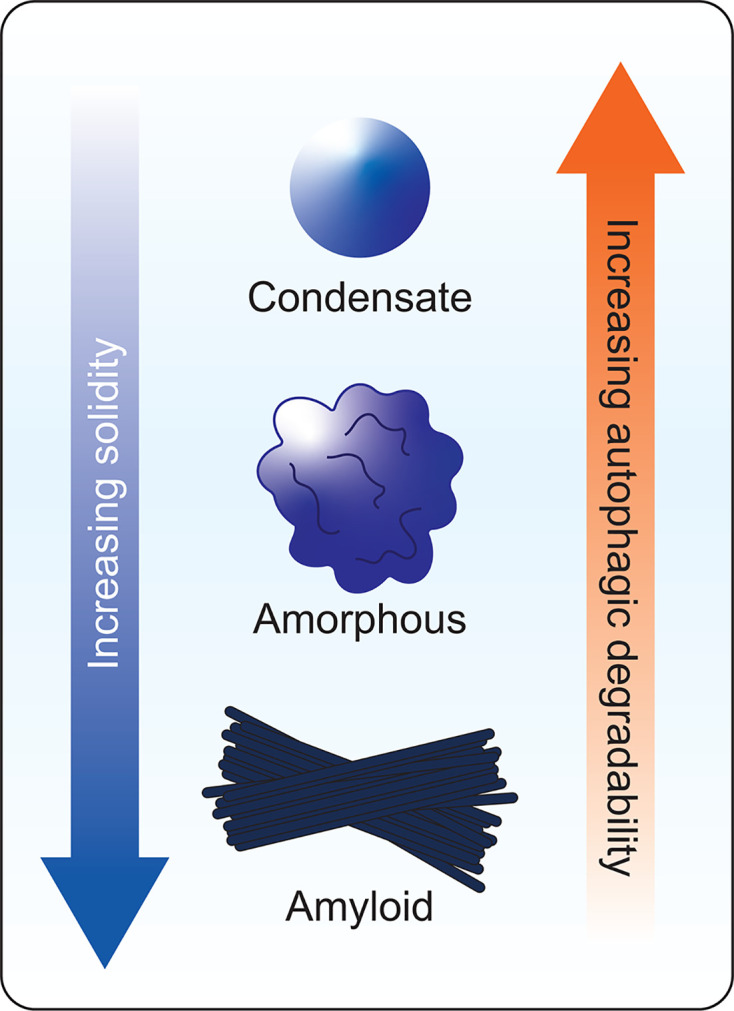
Biophysical states of autophagic cargo Protein aggregates exist as a spectrum of material states, ranging from liquid-like condensates to amorphous aggregates and highly ordered amyloid fibrils, with increasing solidity from top to bottom. Conversely, the capacity of autophagy to degrade less solid, more dynamic cargo is higher and decreases as aggregates become more rigid and ordered.

Protein aggregates are commonly defined as the association of two or more proteins in a non-native conformation [52,53]. Aggregates can vary in size from highly mobile oligomeric forms to micrometer-sized large immobile inclusions. The proteins inside aggregates can form highly ordered amyloid-like fibrils or amorphous structures [54], some proteins can also form both kinds of aggregate, depending on their environment [55–58]. For polyglutamine (polyQ) aggregates, it was shown that both forms can be found in close vicinity to each other [59], or even inside the same inclusion, with fibrillar aggregates forming a radial core region, surrounded by an amorphous aggregate phase [60]. Structures that are morphologically similar to aggregates, are biomolecular condensates. These structures are formed by liquid–liquid phase separation and contain functional proteins. Some phase separated compartments can, over time, undergo a maturation process toward a more solid, aggregate state [61] (Figure 1).

*In* *vitro* fibrillization experiments show that aggregates can be formed from a single protein species. Most isolated fibrils, for which structures have been determined, are also formed by just a single type of protein [62,63], even though some, like α-synuclein filaments from human brains, contain extra densities that cannot be explained by peptides [64]. The picture that emerges from analysis of aggregates in cells and in patient material, however, is more complex. Here, the aggregates often consist not only of fibrils but additionally contain a large number of different proteins, and in some cases, also lipids, nucleic acids and even whole organelles [65–68].

While some aggregates, like heat-shock-induced stress granules, are formed only temporarily and are cleared within hours, others are thermodynamically very stable [10,69,70].

One specific way how cells can eliminate protein aggregates is by a process called disaggregation, which can be followed by refolding of proteins or degradation. Bacteria, yeast, and plants contain specialized chaperones of the AAA+ family, called disaggregases [71], however, direct homologues of this chaperone class have not been identified in multicellular Metazoa. In Metazoa, disaggregation is mainly attributed to the HSP70 machinery. HSP70 can work either in combination with a specific subset of J-domain proteins (JDPs), and HSP110 family nucleotide-exchange factors [72–76] or in combination with the AAA+ chaperone p97/VCP [69,77]. Recently, it has been shown that the effectiveness and substrate fate determination of the HSP70-mediated disaggregation machinery is determined by the amyloid conformation [78,79].

While both metazoan systems are able to disassemble disease-linked amyloid-like fibrils, this process has been reported to result in the generation of seeding-active species that might transmit a disease phenotype to neighboring cells [69,80]. In Metazoa, it is therefore essential to either refold these extracted proteins, or to direct the disassembled species toward degradation and prevent them from reaggregation.

### Aggrephagy: selective autophagic degradation of protein aggregates

**Figure 2:**
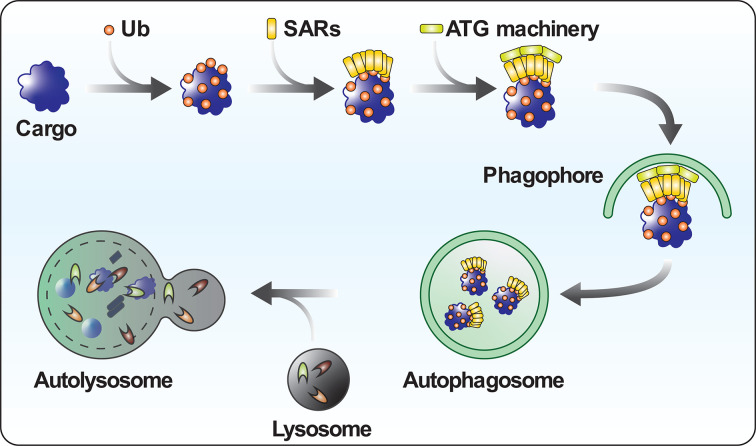
The mechanism of aggrephagy In aggrephagy, degradable cargo is ubiquitylated that enables SARs to bind to and be recruited to the cargo. Recent evidence suggested that it is crucial that the SARs cluster locally on cargo, thereby creating a condensate-like environment that recruits the autophagic machinery, generating an initiation hub. From this initiation hub (or pre-autophagosomal structure; PAS) early autophagosomal membranes (phagophores) form that eventually mature into autophagosomes. Cargo degradation occurs after fusion of the autophagosome with lysosomes.

During selective autophagy cytoplasmic structures are specifically targeted for degradation, including damaged organelles, invading pathogens, large protein aggregates, or other unwanted cellular structures [45,81] (Figure 2). The selectivity of these forms of autophagy is guaranteed by selective autophagy receptors (SARs) that are either membrane-anchored or soluble. Membrane-anchored autophagy receptors are specific membrane proteins of organelles that are activated and/or re-organized upon damage to the respective organelles and subsequently will recruit the autophagy machinery to initiate autophagosome formation [30,81]. In contrast, soluble SARs are not integrated in membranes but have the ability to bind their respective cargo either directly or indirectly by binding to ubiquitin moieties that were previously attached by specific E3-ligases [30] using dedicated ubiquitin-binding domains (e.g. UBA/UBAN or related modules) [31]. Besides cargo binding, soluble SARs link cargo to the ATG machinery by binding ATG8 proteins (LC3/GABARAP) via LC3-interacting regions (LIRs) and, in many cases, FIP200 via FIP200-interacting regions [82,83]. Selective autophagy, bulk autophagy, and other forms of autophagy (e.g. microautophagy) are not mutually exclusive and can influence each other. During the first hour after starvation-induced bulk autophagy, a selective set of SARs (e.g. p62/SQSTM1, NBR1, TAX1BP1, NDP52, and NCOA4) are degraded via microautophagy, possibly providing a regulatory feedback mechanism [84].

In aggrephagy, the decision whether an aggregate becomes a degradable substrate is not dictated by ubiquitin alone, but rather determined by multiple factors: aggregate architecture (e.g. amorphous or amyloid aggregates; p62 bodies/aggresomes), cargo state (liquid/gel/solid), surface chemistry (ubiquitin density/linkage and accessibility of autophagy machinery), and mechanical and/or size constraints that determine whole-particle engulfment versus piecemeal removal (Figure 1). These features help explain why upstream remodeling steps that alter size, accessibility, and material properties of aggregates can be rate-limiting for efficient clearance. In aggrephagy, soluble SARs such as SQSTM1/p62, TAX1BP1, and NDP52 connect ubiquitylated aggregates to the autophagy machinery by engaging FIP200 and ATG8-family proteins [31,81,85]. This SAR–cargo interaction is further regulated by TBK1 (TANK-binding kinase 1)-dependent phosphorylation, which can enhance SAR binding to Ub, FIP200 and LC3/ATG8 proteins [30].

Some particular SARs, i.e. NBR1 and p62, have also sequestrase-like functions, besides their role in marking cargo for degradation. Both proteins contain a PB1 (Phox and Bem1) domain, which enables oligomerization, leading to higher-order filaments that, together with ubiquitin binding, can promote clustering of ubiquitylated proteins into condensate structures. Subsequent recruitment of other SARs, such as TAX1BP1, NDP52, and optineurin (OPTN), can then promote cargo degradation by coupling these assemblies to the autophagy machinery [30,86–89].

Aggrephagy of certain cargo can additionally be facilitated by autophagy receptors and co-factors that do not belong to the canonical SARs, e.g. CCT2, ALFY, HSPB7, or BAG3. The TCP1 ring complex (TRiC) subunit CCT2 has been proposed as a stand-alone, ubiquitin-independent receptor with a preference for more solid, amyloid-like assemblies that works independently from the TRiC complex [31,90]. The adaptor protein ALFY facilitates the lysosomal clearance of cargo [31,91,92]. ALFY is recruited to ubiquitylated aggregates via p62/NBR1, to promote cargo sequestration to p62 bodies, and to scaffold local autophagosome biogenesis by linking cargo to ATG proteins and autophagosomal membranes [31,43]. The small heat shock protein HSPB7 and the co-chaperone BAG3 primarily facilitate substrate handling and clustering rather than serving as receptors per se. HSPB7 keeps newly synthesized aggregation-prone proteins like polyQ-extended forms of Huntingtin Exon 1 (Htt-polyQ) in an autophagy-accessible state but does not resolve pre-existing aggregates [93,94]. BAG3 participates in CASA/BIPASS (chaperone-assisted selective autophagy/BAG3-instructed proteasomal to autophagosomal switch and sorting) and can shift HSP70-bound, ubiquitylated substrate proteins (e.g. Htt-polyQ, ALS-associated SOD1 mutant proteins, poly-ubiquitylated filamin proteins, and Tau) from proteasomal to lysosomal degradation during aging or proteasome stress [95–99]. Together, this diversity of aggrephagy factors highlights the redundancy and flexibility of this system to be able to deal with different cargo species in different environments (e.g. tissue-specific expression profiles of receptors [100]).

In recent years, two important aspects for SAR-mediated cargo degradation beyond the stochastic binding to ubiquitin emerged: SAR clustering and SAR liquidity and mobility [101,102] (Figure 2). The clustering of SARs has been shown to play an important role in ER-phagy, mitophagy, and aggrephagy [103–107]. This clustering generates condensate-like features that keep the SARs mobile on the cargo [102]. The mobility of SARs is essential for several kinds of selective autophagy, such as mitophagy, lysophagy, or ER-phagy [108,109] and also for the degradation of p62 bodies or budding yeast Ape1–Atg19 condensates [102]. As a result, SAR clustering leads to the local concentration of autophagy machinery components (e.g. FIP200, ATG9 in mammals, and Atg11 in yeast) that are recruited to the cargo and phase separate themselves [108,110], generating autophagosome initiation hubs [102]. Such condensation events around cargo (also called wetting) are essential for the formation of the autophagosomal membrane by the ATG machinery [111]. Interestingly, the liquidity of p62 during lysophagy is maintained by the interaction with the sHSP27 [112], highlighting the interplay between the different PQC systems.

### Aggregate remodeling for aggrephagic degradation

SAR clustering is an important feature for the initiation of selective forms of autophagy, however, it is not fully understood how such clustering is achieved. For different forms of selective autophagy of organelles (organellophagy), efficient turnover depends on degrading organelles in smaller sections rather than as a single unit. This is often achieved by prior fragmentation of organelles, which is likely the mechanism that leads to the local clustering of SARs [30,103,104]. Prominent examples of organelle fragmentation as a trigger for selective autophagy are fragmentation of mitochondria [113–115], peroxisomes [116], or the Golgi apparatus [117,118]. Piecemeal autophagy has also been described for parts of the nucleus (nucleophagy) [119,120], the endoplasmic reticulum [103,104,121], or in plant cells the chloroplast [122]. This highlights that for efficient selective autophagosomal cargo degradation, cargo-specific remodeling systems prime cargo before the selective autophagy machinery captures them and initiates degradation.

**Figure 3:**
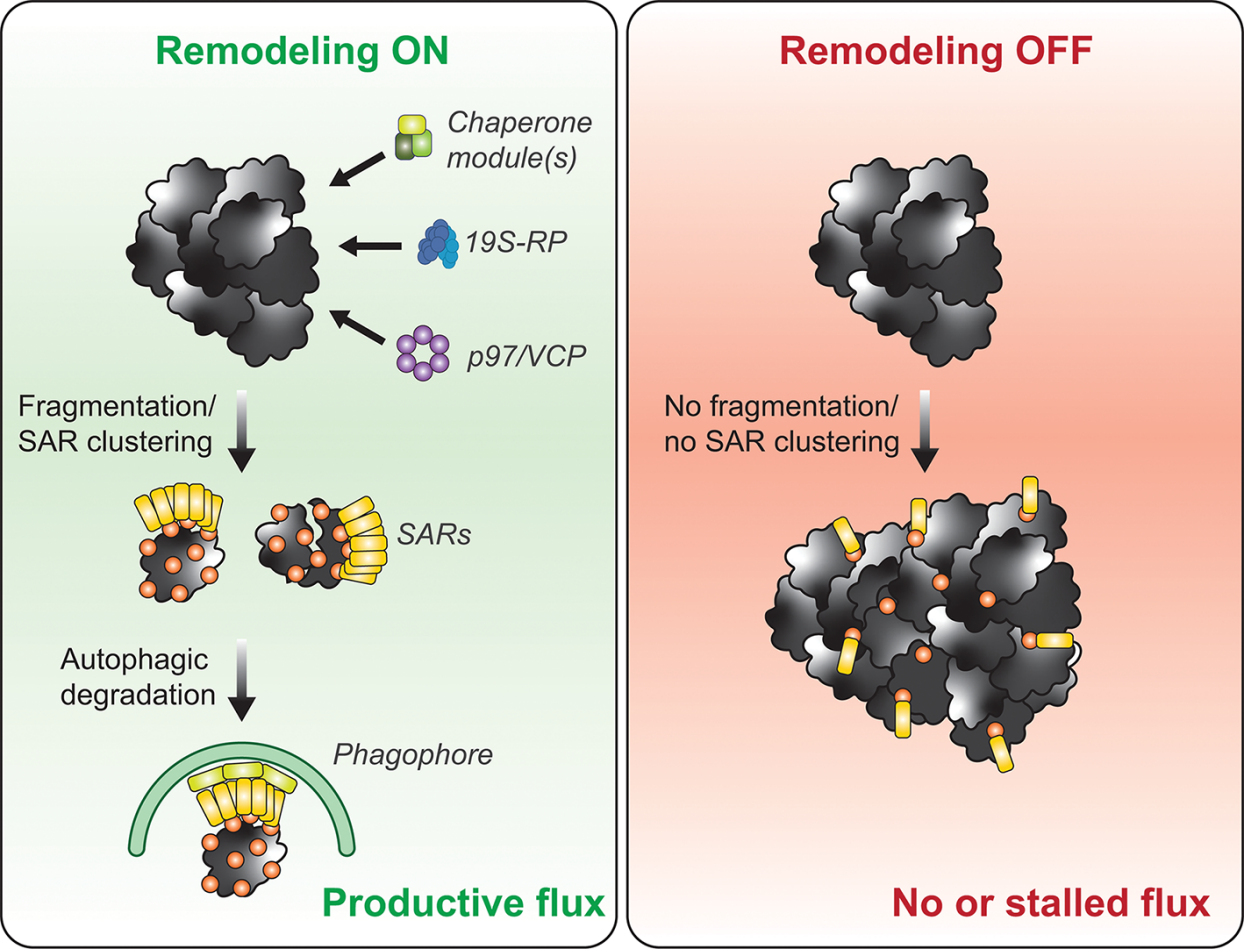
Cargo remodeling is required for autophagic degradation Prior to efficient aggrephagic degradation, cargo needs to be remodeled and fragmented by cellular remodeling machineries. Components of these machineries are chaperones (e.g. DNAJB6, HSP70, HSP110) and AAA+ ATPases (e.g. p97/VCP, 19S-RP). Remodeling and fragmentation of the protein aggregates enables SAR clustering and subsequent aggrephagic degradation ensuring a productive degradation flux. In the absence of this remodeling/fragmentation step, no SAR clustering occurs and the degradation flux is absent or stalled.

Recently, two studies demonstrated that piecemeal autophagy and/or the fragmentation of aggregates is also required for aggrephagy [107,123] (Figure 3). The fragmentation of organelles prior to selective autophagy is often mediated by the organelle’s fission machinery, as shown in the context of mitophagy [124,125] or pexophagy [116], however, in the context of aggrephagy, where there is no cargo-intrinsic fission/fragmentation machinery, cells have to employ alternative strategies. Cells repurpose or ‘borrow’ AAA+ ATPases and chaperone/proteasome modules to generate such degradable fragments. An emerging theme in aggrephagy is the requirement to process aggregated structures (e.g. aggresomes or amorphous inclusions) by cellular fragmentation or disaggregation machineries prior to their lysosomal removal [101,107,123]. One remodeling route centers around p97/VCP, an AAA+ ATPase, which is required to disintegrate aggregates and thereby enables piecemeal aggrephagy [123]. Inhibition of p97/VCP left aggregates largely ‘intact’ and incapable of being incorporated into autophagosomes even though key autophagy factors and SARs (including p62/SQSTM1, TAX1BP1, and WIPI2) were present [123]. A second remodeling route entails a chaperone module consisting of DNAJB6–HSP70–HSP110 that works together with the proteasomal 19S-RP to fragment diverse amorphous aggregates as a prerequisite for their autophagic clearance [107]. Notably, the 19S-RP, similar to p97/VCP, contains AAA+ ATPase activity that is classically coupled to the translocation of substrates into the 20S core particle (20S-CP) of the proteasome for proteolysis. For this described fragmentation activity, the 19S-RP appears to work independently of the 20S-CP proteolytic capacity [107]. These findings further highlight the close connections between PQC systems, which can cooperate but also regulate each other (e.g. CASA/BIPASS [126] or proteophagy [127]). Consistent with the p97/VCP-mediated aggregate removal by piecemeal autophagy, it was observed that when fragmentase components (e.g. DNAJB6 or 19S-RP) are depleted, the aggregates were not cleared despite the presence of SARs and other ATG proteins (including NDP52, TAX1BP1, FIP200) [107,123]. These findings emphasize that mere recruitment of SARs and ATG proteins to cargo is not sufficient for degradation, but requires remodeling of aggregates for successful aggrephagy (Figure 3). Although not experimentally proven, it is tempting to speculate that the fragmentation and compaction of large inclusions into smaller pieces leads to the crowding and therefore clustering of the SARs [107]. It is also possible that the biophysical architecture of the larger aggregates inhibits the avidity of the SARs, which is required for their clustering [102,105]. Interestingly, DNAJB6, a co-chaperone of HSP70 that is known to suppress amyloid formation of multiple neurodegeneration-associated proteins [128–132], plays a role in both remodeling routes and appears to be required for efficient autophagic degradation [107,123]. In contrast, p97/VCP is dispensable for the DNAJB6–HSP70–HSP110–19S-RP route [107], suggesting that both routes are mechanistically distinct. So far, p97/VCP or DNAJB6–HSP70–HSP110–19S-RP have been shown in cellular systems to work on aggresomes, fibrillar tau aggregates, or on amorphous aggregates, such as the chemical-inducible PIM aggregates, puromycin-induced aggregates or pre-amyloid huntingtin aggregates, respectively [69,107,123]. Future studies will show whether other aggregate species are also targeted by these remodeling routes, especially considering the fact that some fibrillar aggregates like Htt-polyQ, are not recognized by the autophagy machinery [60].

Aggregate fragmentation, however, is not always beneficial and can be considered a double-edged sword. *In vitro* incubation of amyloid fibers with cellular disaggregation machineries (HSP70, HSP110, and certain JDPs, such as DNAJB1, DNAJA1, or DNAJA2) can generate seeding-competent fibrillar fragments [69,80], likely determined by the amyloid conformation [78,79]. When introduced into cells or animals, such fragments are poorly cleared and induce cellular or organismal toxicity [133]. These observations highlight that cells harbor multiple disaggregation/fragmentation activities with distinct substrate preferences, and it remains to be determined why fragmentation of some aggregate species enables productive clearance whereas other fragments become toxic. In addition, it is possible that the efficiency of the fragmentation–degradation axis depends on the presence of certain fragmentation components, such as DNAJB6 that can prevent the amyloid cascade when incubated together with amyloid-prone proteins [128], but is unable to work on already formed amyloids [134].

Altogether, these recent studies support a model that suggests that remodeling of aggregates is a prerequisite for aggrephagy. In both systems, the p97/VCP-mediated aggregate removal [123] and the DNAJB6–HSP70–HSP110–19S-RP fragmentation pathways [107], aggrephagy is impaired when the remodeling is inhibited despite the presence of SARs and the autophagy machinery present at the cargo. Hence, recruitment of the autophagy machinery alone is insufficient for degradation, but requires a remodeling of at least some aggregate species into ‘aggrephagy-competent’ cargo units.

## Perspectives

- Selective autophagy is an important part of the PQC system and is crucial for the clearance of protein aggregates. Aggregate clearance does not only depend on the presence of autophagy factors but also on their clustering.
- Remodeling of selective autophagy cargos, including protein aggregates, is required for efficient autophagic clearance. Amorphous aggregates are more efficiently targeted by autophagy when compared to fibrillar structures.
- Research in the next years needs to focus on the interplay of the different parts of PQC (e.g. UPS, autophagy, chaperones) and not only study them in isolation. Answering the question which properties of an aggregate (size, biophysical properties, or other yet unknown features) will allow it to be handled in the most beneficial manner for individual cells, but also cells in the vicinity, should inform the development of aggregation-modulating interventions, and presents an essential step for the development of treatments for protein aggregation diseases.

## Open Access

Open access for this article was enabled by the participation of the University of Groningen in an all-inclusive Read & Publish agreement with Portland Press and the Biochemical Society.

## Abbreviations

19S-RP: 19S regulatory particle
20S-CP: 20S core particle
ATG: autophagy-related
CMA: chaperone-mediated autophagy
JDP: J-domain protein
LIR: LC3-interacting region
PolyQ: polyglutamine
PQC: protein quality control
SAR: selective autophagy receptor
TRiC: TCP1 ring complex
UPS: ubiquitin–proteasome system
